# Identification and verification of potential piRNAs from domesticated yak testis

**DOI:** 10.1530/REP-17-0592

**Published:** 2017-11-03

**Authors:** Jishang Gong, Quanwei Zhang, Qi Wang, Youji Ma, Jiaxiang Du, Yong Zhang, Xingxu Zhao

**Affiliations:** Gansu Agricultural UniversityLanzhou, People’s Republic of China

## Abstract

PIWI-interacting RNAs (piRNA) are small non-coding RNA molecules expressed in animal germ cells that interact with PIWI family proteins to form RNA–protein complexes involved in epigenetic and post-transcriptional gene silencing of retrotransposons and other genetic elements in germ line cells, including reproductive stem cell self-sustainment, differentiation, meiosis and spermatogenesis. In the present study, we performed high-throughput sequencing of piRNAs in testis samples from yaks in different stages of sexual maturity. Deep sequencing of the small RNAs (18–40 nt in length) yielded 4,900,538 unique reads from a total of 53,035,635 reads. We identified yak small RNAs (18–30 nt) and performed functional characterization. Yak small RNAs showed a bimodal length distribution, with two peaks at 22 nt and >28 nt. More than 80% of the 3,106,033 putative piRNAs were mapped to 4637 piRNA-producing genomic clusters using RPKM. 6388 candidate piRNAs were identified from clean reads and the annotations were compared with the yak reference genome repeat region. Integrated network analysis suggested that some differentially expressed genes were involved in spermatogenesis through ECM–receptor interaction and PI3K-Akt signaling pathways. Our data provide novel insights into the molecular expression and regulation similarities and diversities in spermatogenesis and testicular development in yaks at different stages of sexual maturity.

## Introduction

The yak (*Bos grunniens*) is one of the most remarkable domestic animals and an iconic symbol of the Qinghai-Tibetan plateau. Tibetans and other nomadic pastoralists depend on yaks in high altitude environments ranging from 3000 to 5500 m above sea level. There are 1.3 million yaks in China, making up 90% of the world’s population. The limitations of plateau environments, the underdeveloped economy and limitations of scientific approaches have limited the scientific study of yaks compared to other domesticated livestock. The recent completion of the yak genome sequencing (Qiu *et al.* 2012) provides an actionable ‘blueprint’ for understanding various aspects of yaks, such as breeding, environmental adaptation and reproductive performance. Most of the genes in the yak genome have been annotated based on bioinformatics predictions. However, much information remains incomplete, especially related to the reproduction and development of yak. Reproduction is the key to improving the performance of yaks. In particular, the improvement of the male yak breeding stock cannot be neglected in yak-producing areas. Therefore, it is of practical significance to understand and to master the reproductive and physiological characteristics of yaks.

PIWI-interacting RNAs (piRNAs) are a class of newly discovered small non-coding RNAs (ncRNAs) that are approximately 24–32 nucleotides (nt) in length, and defined by their specific binding to the PIWI subfamily of the ARGONAUTE (AGO)/PIWI family of proteins (Girard *et al.* 2006). Similar to microRNAs, the 5′ends of piRNAs exhibit a strong uracil bias (Aravin *et al.* 2006*b*). piRNAs play roles in the physiological regulation of germline development and spermatogenesis, genome protection from transposons and regulation of mRNAs and long non-coding RNAs (Watanabe *et al.* 2015). The *PIWI* clade is present in all animals, and these proteins are most abundantly expressed in the germline (Carmell *et al.* 2002). The piRNAs originate from transposons and repetitive sequences of germ cells and are expressed in the gonads (Liu & Paroo 2010, Siomi *et al.* 2011*a*, Pillai & Chuma 2012), suggesting a role in germline stem cell maintenance including restricting the expression of transposable elements. *PIWI* mutations in *Drosophila* (Aravin *et al.* 2001), *C. elegans* (Stower 2012), mice (Pillai & Chuma 2012) and Zebrafish (Houwing *et al.* 2007) caused gametogenic defects such as failure of germline establishment, loss of germline stem cells, meiotic arrest and blockage in spermiogenesis, leading to sterility (Juliano *et al.* 2011). However, little is known about the diversity of piRNAs, and their regulatory contribution in domesticated animals, particularly in yaks.

Normal testis and spermatogenic development has a vital role in species breeding, making this research important to improve the semen quality and to promote livestock production. Additionally, piRNAs play an important role in various stages of testicular tissue development and spermatogenesis, though this has been shown only in animal models and in humans. Overall, the specific functions and mechanisms of piRNAs remain unknown. In the present study, we analyzed, using RNA-Seq technology and described the piRNAs from domesticated yak testis at different stages of sexual maturity. This is the first report regarding the complexity of the piRNA transcriptome during the essential processes of spermatogenesis and the results reveal the predominant role of piRNAs in limiting transposon proliferation in yak testis.

## Materials and methods

### Animal sampling

We prepared six samples of total RNA from yak testes using a purification kit from Ambion (Thermo Fisher Scientific). Samples were obtained from six individual male black yaks (Qinghai Plateau Yak), with ages ranging from <2 to 8 years old, at a slaughterhouse in Qinghai province (China) during July and August. Male yaks appear to be sexually mature at age five years and according to the dental age of the yaks (Mohanty *et al.* 1999), the animal samples were divided into two groups. A were sexually immature (sample 1–3 is 1–2, 2–3 and 3–4 years old, respectively), while the B group were sexually mature animals (sample 1–3 is 5–6, 6–7 and 7–8 years old, respectively). Genomic DNA was isolated from tissue samples from four different age stages (<2, 2–4, 4–6 and 6–8 years) for validation testing. This study was supervised by and all experiments approved by the Animal Care Commission of College of the Veterinary Medicine, Gansu Agriculture University, China.

### Reagents

The TransZol UP RNA extraction kit and TranStart Tip Green qPCR SuperMix were acquired from TransGen Biotech (Beijing, China), and diethyl pyrocarbonate and DEPC were purchased from Beyotime (Beijing, China). Passive Reference Dye and RevertAid First Strand cDNA Synthesis Kit were procured from Thermo Fisher Scientific. Argonaute2 (Bs-20459R) and Argonaute3 (Bs-12517R) antibodies were purchased from Bioss (Beijing, China) and the immunohistochemical kit (sp-002) and DAB (3,3N-diaminobenzidine) chromogenic reagent kit were acquired from Solarbio Life Sciences (Beijing, China). All reagents and kits were used as received without modification.

### The method

#### piRNA sequencing

The cDNA samples obtained from total RNA were sequenced on an Illumina HiSeq 2500 machine using the single-end 50 bp format. The sequences have been deposited into the NCBI short read archive under study number (SRP021475).

The adapters were computationally stripped off the raw sequencing reads using the Cutadapt tool, keeping the reads between 18 and 44 bp in length after stripping.

The linkers were removed from the obtained clean data and sequences of low quality were removed. The resulting sequences were used to BLAST the Rfam database, v11 (Burge *et al.* 2013) and miRbase database release 21 (Ana Kozomara 2014) to determine the sources of the reads. Redundant sequences, including rRNA, tRNA, snRNA and snoRNA were removed. To identify the remaining short RNA sequences, a blastn-short search was performed.

The reads were aligned to the yak reference genome (fmt.bgr_ref_BosGru_v2.0_chrUn.fa), which annotates yak reference repeat regions of the genome, downloaded from NCBI. Because more piRNA sequences are related to transposable element (TE) (Brennecke *et al.* 2008, Siomi *et al.* 2011*a*). We annotated the TEs in the yak reference genome using RepeatMasker version open-4.0.1, RepBase Update 20120418, RM database version 20120418 and a default mod run with RMBLASTn version 2.2.27^+^. Reads mapped to known non-coding RNA genes (miRNAs, rRNAs, snRNAs, snoRNAs, tRNAs in Ensembl, and RNA repeats identified in RepeatMasker) were removed from the datasets. The remaining reads were mapped to the yak reference genome using Bowtie (version 1.1.2) allowing up to 2 mismatches and multiple matches (-a –best –strata -v 1). Reads that are mapped to the assembled chromosomes in the reference genome and were between 24 and 35 nt were selected as putative piRNAs. Previous work (Castellano *et al.* 2015) has shown that piRNAs are found in clusters throughout the genome that may contain as few as ten or up to many thousands of piRNAs and can vary in size from one to one hundred kb (O’Donnell & Boeke 2007). piRNA clusters were identified from putative piRNAs based on Poisson’s distribution of random distribution in the yak genome. Specifically, we slid a 2 kb window by 400 bp steps along the genome and counted the normalized number of piRNAs in each window using the RPKM metric. We included piRNAs that mapped to multiple positions in the genome, but divided the number of these multiple-mapping piRNA reads by the number of mapping positions to estimate their relative abundance (e.g., a piRNA that mapped to 10 positions was counted as 0.1 reads at each position).

#### GO enrichment and gene pathways analyses

The DAVID functional annotation tool (http://david.abcc.ncifcrf.gov/) was used to perform GO classification and pathway annotation of piRNA-generating mRNAs (Huang *et al.* 2008). Functional annotation terms from the ontologies of ‘biological processes’ and ‘molecular function’ were recorded with an EASE threshold set to 0.1 and count threshold of 2 (Sun *et al.* 2011). The enrichment score cutoff was set to 1.0. The resulting protein sequences of the genes identified from the GO analysis were mapped to the STRING database (http://www.string-db.org/) and used to construct a protein–protein interaction (PPI) network.

#### Phylogenetic analyses

Amino acid and nucleotide sequences for the PIWI subfamily of AGO/PIWI family proteins of 12 species were obtained from the National Center for Biotechnology Information database (http://www.ncbi.nlm.nih.gov). Phylogenetic analysis was carried out by the Neighbor-Joining statistical method (Saitou & Nei 1987) using MEGA5 software, version 6.06 and the MUSCLE algorithm (Edgar 2004). To improve the phylogenic tree, we omitted partial and repetitive sequences of the same species.

#### qPCR analysis of piRNAs and mRNAs

Total RNA was extracted from 4 different age groups of testis tissue using the TransZol UP RNA kit according to the manufacturer’s instructions. After treatment with DNase, RNA was subjected to reverse transcription using a RevertAid First-Strand cDNA Synthesis Kit according to the supplier’s instructions. qRT-PCR and RT-PCR were carried out as reported previously (Zhang *et al.* 2014).

qPCR was performed to examine the expression of 3 repeat sequence-linked putative piRNAs and 14 genic transcript-linked putative piRNAs, along with their 12 linked genes, in immature and mature testis. Total RNA was isolated from samples using TRIzol reagent. For piRNA amplification, 1 μg total RNA was reverse transcribed using a *U6* snRNA and miRNA first-strand cDNA synthesis kit (Genepharma, Suzhou, China). To elongate the piRNA, total RNA was treated with *Escherichia coli* poly-A polymerase to generate a poly-A tail at the 3′-end of each RNA molecule. Following polyadenylation, cDNAs were synthesized using the RT adaptor primer. Quantitative real-time PCR was carried out using the Hairpin-it microRNA and *U6* snRNA Normalization RT-PCR Quantitation Kit (Genepharma). The PCR mixture was prepared by adding 10 μL of 2× Real-time PCR Master Mix, 0.4 μL of 10 μM miRNA/*U6* snRNA specific primer set, 2 μL of 50× ROX reference dye, 0.2 μL of Taq DNA polymerase (5 U/μL), and 2.0 μL of miRNA RT product to a final volume of 20 μL. PCR was performed with an initial incubation at 95°C for 3 min, followed by 40 cycles at 95°C for 12 s, 62°C for 40 s, 72°C for 30 s and a final extension of 1 cycle for 5 min at 72°C.

The published sequences of the yak *AGO2* and *AGO3* genes and *PIWI-like 1–4* genes were used to design primer sequences (Supplementary Table 1, see section on supplementary data given at the end of this article) using Primer 5.0 software (Premier Biosoft International, Palo Alto, CA, USA). The primers were synthesized by Shanghai Sangon Biological Company (Shanghai, China).

### Western immunoblot and immunohistochemistry

Western blot and immunohistochemical staining were carried out as previously reported (Mcclusky *et al.* 2009). The immunohistochemical sections were observed and photographed using an Olympus-71 optical microscope. The cells were counted by Image-Pro Express computer-aided image analysis software. Three CIRP immunohistochemical sections from different age groups were selected. Five sections of 0.148 mm^2^ were randomly selected (about 8–400× magnification fields). In each field of view, five seminiferous tubules were selected to determine, the ratio of CIRP-positive cells.

### Statistical data

All experimental data were presented as mean ± standard error of mean (s.e.m.). The statistical significance of differences, in mRNA and protein expression of *AGO2* and *AGO3* in the different testis groups were assessed using SPSS20.0 software (IBM) by one-way ANOVA followed by Tukey’s Honestly Significant Differences (HSD) for multiple comparison. Differences were considered significant if *P* < 0.05.

## Results

### Sequencing of yak piRNAs in sexually mature and immature stages

**Figure 1:**
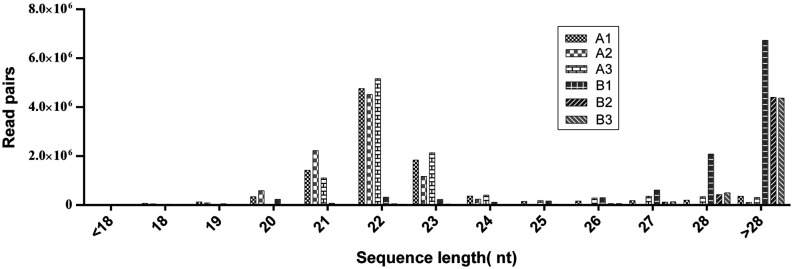
The length distribution of miRNA and piRNA in samples from sexually mature yak testis. The small RNAs of yak testis displayed a bimodal length distribution with two peaks at 22 nt (miRNA) and >28 nt (piRNA). The miRNA was enriched in the sexually immature group; however, the piRNA was more abundant in the sexually mature group.

To identify yak piRNAs, sequencing was performed on two groups of testis, from sexually immature (A group) and sexually mature (B group) animals (Table 1). To characterize the piRNAs, we first performed deep sequencing on the small RNAs ranging from 18 to 40 nt in length and identified 4,900,538 unique reads from a total of 53,035,635 reads (Table 1). The raw reads from both groups were annotated and classified based on length. The yak miRNAs exhibited a bimodal length distribution with two peaks at 22 nt and >28 nt (Fig. 1). Sequence analysis of cloned small RNAs with a length of 18–44 nt indicated that 70% contained a 5′ uridine residue (Supplementary Fig. 1). In total, we obtained 4,637,597 unique reads from the six samples. We then removed reads that matched known small RNAs (e.g., rRNA, tRNA, snRNA and snoRNA), reads that did not map to the reference genome and reads that were outside of the piRNA size range, leaving 3,106,033 reads that were considered putative piRNA reads. The unique putative piRNA sequences were subjected to additional analysis.

**Table 1:** Summary of the number of reads at each processing.

**Sample**	**A1**		**A2**		**A3**		**B1**		**B2**		**B3**		**Total**	
	Total	Unique	Total	Unique	Total	Unique	Total	Unique	Total	Unique	Total	Unique	Total	Unique
Raw reads	10,761,968	757,571	9,609,790	383,104	10,796,435	852,959	10,851,647	1,226,473	5,615,429	872,106	5,400,366	808,325	53,035,635	4,900,538
Remove known small RNAs	9,990,121	704,131	9,069,035	283,120	10,638,193	823,634	10,664,043	1,199,285	5,192,139	839,987	5,196,233	787,440	50,749,764	4,637,597
Remove unmapped reads to the reference genome	7,776,826	428,435	8,097,634	78,436	7,067,642	561,284	9,664,336	971,660	4,749,994	673,142	4,767,914	637,818	42,124,346	3,350,775
Putative piRNA	332,457	248,369	26,912	15,146	474,028	368,048	918,545	714,687	691,568	516,576	662,523	495,678	3,106,033	2,358,504

A (1–3) Yak sexually immature testis; B (1–3) Yak sexually mature testis.

### BLAST alignment of piRNAs to yak genome

The reads were mapped to the yak reference genome to infer putative piRNA sequences and identify piRNA clusters (that presumably overlap significantly with piRNA) using bowtie (Hassall & Burgess-Limerick 2016). The putative piRNAs reads with lengths of 18–40 nt (3,106,033) were chosen for yak genome assembly using the Short Oligonucleotide Analysis Package (SOAP), leading to 11,187,432 genome-matched reads (Supplementary Table 2). Only sequences that perfectly matched the yak genome along their entire length were considered for further analysis. Consequently, the genome-matched reads for 21.09% (11,187,432) of total reads (53,035,635), which were perfectly mapped to 10,125 locations in the draft assembly of the *Bos grunniens* genome. Previous work showed that piRNAs are found in clusters throughout the genomes, which may contain as few as ten or up to many thousands of piRNAs, varying in size from one to one hundred kb (O’Donnell & Boeke 2007). To identify these clusters, we slid a 2 kb window along each chromosome in 0.4 kb along and counted the number of piRNAs in each window as Reads Per Kilobase per Million mapped piRNAs (RPKM; see ‘Methods’ section for details). Using a relaxed RPKM cutoff of one, we identified 331 cluster regions ranging in size from 2 kb to 69 kb, with over 80% of the putative piRNAs within one of the clusters. We identified 6388 candidate piRNAs from clean reads. Of these, candidate piRNAs, 75% start with a U (Supplementary Fig. 2).

### Classification of the piRNA function

**Figure 2:**
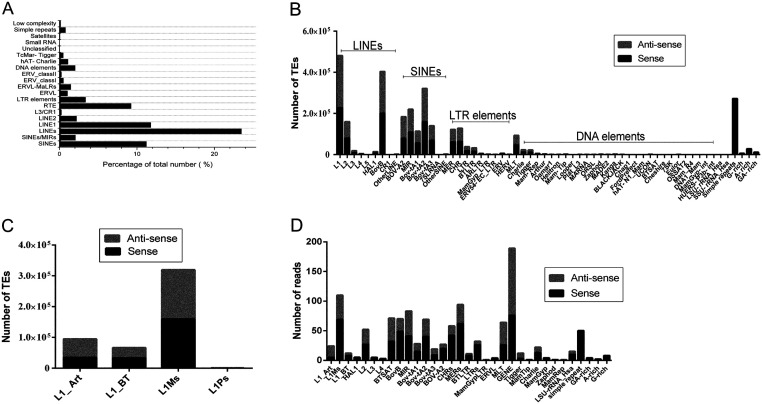
Repetitve elements in the yak piRNA sequences identified by RepeatMasker. (A) piRNAs were mapped against *Bos grunniens* genomic transposable elements (TEs). (B) Results show the classes of TEs represented in the *Bos grunniens* genomic in both the sense (black) and antisense (grey) orientations. (C) Results indicate that the classes of L1 represented in the *Bos grunniens* genomic in both the sense (black) and antisense (grey) orientations. (D) Results from the testis show the classes of TEs represented in the piRNA populations in both the sense (black) and antisense (grey).

About 23.48% (730,493) and 3.36% (163,507) of the yak genome derived from long interspersed nuclear elements (LINEs) and long terminal repeat elements (LTR_elements), respectively (Fig. 2A). Other sequences were identified as short interspersed nuclear elements (SINEs), 11.21% (1,079,801), DNA elements, 2.02% (143,851), unclassified, 0.02% (1607), total interspersed repeats, 40.09%, small RNA, 0.01% (1467), satellites, 0.06% (405), simple repeats, 0.78% (271,421) and low complexity, 0.15% (44,321). The LINEs, SINEs and simple repeats are the most abundantly represented transposable element (TE) classes in the yak genome, representing over 50% of the mapped genome sequences (Fig. 2A). Within the LINE_L1 classes, the genome mapped predominantly to L1_Art repeats in the antisense direction (Fig. 2B and C). The average number of BovB in both stands was comparable. Of the SINEs, highly represented TEs are the MIR, BOV-A2 and Bov-tA elements, all of which are short, non-autonomous repeats. Interestingly, the DNA TE Tigger and Charlie were substantially represented, with >99% of the piRNAs associated with the antisense strand. Previous work showed that piRNAs are mainly concentrated in the TE and intergenic regions (Ku & Lin 2014), with few piRNAs distributed in exon regions. Candidate piRNAs were mapped to the bovine TEs annotated by Repeat Masker to determine their potential target transcripts.

Our analysis indicated that different piRNAs have different functions, so the piRNAs were categorized according to function. Our analysis revealed 189 gene type piRNAs and 964 TE-type piRNAs, including 76 sense gene type, 113 antisense gene type, 590 sense TE-type and 374 antisense TE-type (Supplementary Table 3). Interestingly, most of the piRNAs mapped predominantly to L1_Art repeats in the antisense direction (Fig. 2D).

### Gene ontology (GO) term analysis

**Figure 3:**
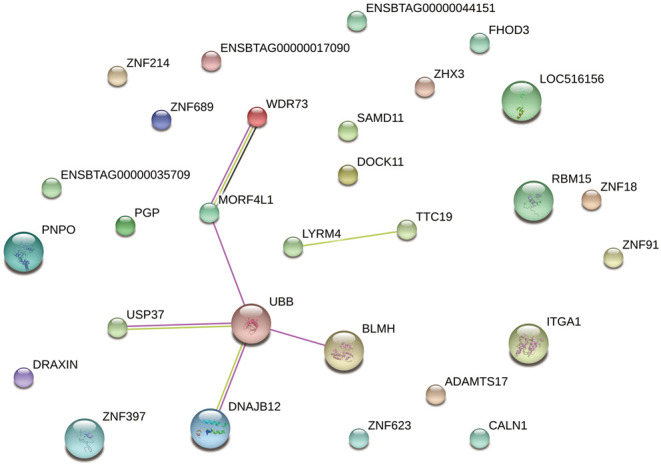
The analysis of PPI interaction network. A total of 6 pairs of protein interactions with reliability score greater than 0.9 were selected, and 29 nodes accounted for germ cell development.

GO enrichment analysis revealed that the biological processes involved mainly included cellular component organization or biogenesis, cellular process, developmental process, multicellular organismal process, single-organism process, biological adhesion, multi-organism process, growth, reproductive process and rhythmic process (Supplementary Fig. 3). KEGG pathway analysis showed that piRNA-generating genes played important roles in 26 pathways, including vitamin B6 metabolism, protein processing in the endoplasmic reticulum, Parkinson’s disease, glyoxylate and dicarboxylate metabolism, carbon metabolism, thyroid hormone signaling pathway, PI3K-Akt signaling pathway, focal adhesion, ECM–receptor interaction and hematopoietic cells (Supplementary Table 4). Overall, most of the gene pathways were associated with metabolism. The protein sequences of the genes from the GO analysis were mapped to the STRING database (http://www.string-db.org/) and used to construct a protein–protein interaction network. Six pairs of protein interactions with reliability scores greater than 0.9 were selected, and 29 nodes accounted for germ cell development. As shown in Fig. 3, the protein interaction network of these genes presented a lowly aggregated state. Eight proteins (UBB, USP37, DNAJB12, BLMH, MORF4L1, WDR73, LYRM4 and TTC19) showed strong interactions with each other. In particular, UBB had several strong interactions, suggesting activity in the nucleus (GO: 0005634), cytoplasm (GO: 0005737) and male meiosis (GO: 0007141).

### Expression analysis of putative piRNAs and piRNA-linked genes

**Figure 4:**
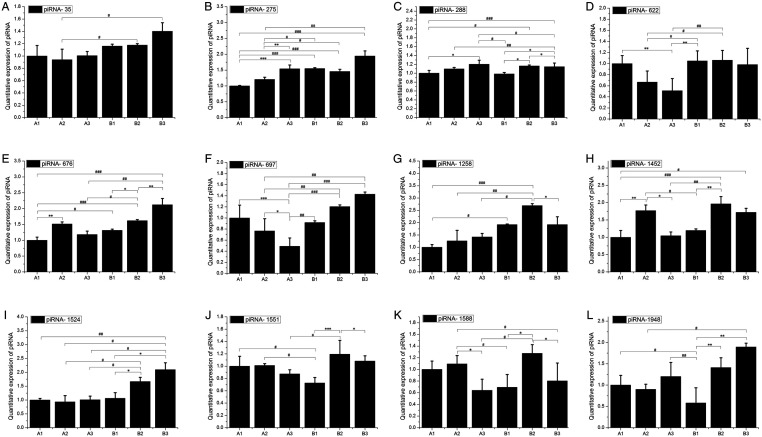
qPCR analysis of piRNAs. qPCR quantitative expression analysis of 14 genic-transcript-linked piRNAs that were upregulated in group B, along with their linked coding genes. cDNA templates used for the amplification of piRNAs and genes were prepared separately, and amplified with appropriate piRNA and gene-specific primers. The expression of piRNAs and genes were normalized against the levels of snoRNA and *U6*, respectively. (A, B, C, D, E, F, G, H, I, J, K and L) Represents piRNA (35, 275, 288, 622, 676, 697, 1258, 1452, 1524, 1551, 1588 and 1948, respectively. Bars indicate the s.e.m. of triplicate analyses. ^#/*^P < 0.05, ^##/**^P < 0.01 and ^###/***^P < 0.001.

To confirm the expression of piRNAs identified by sequencing, we conducted RNA adapter-based qPCR on sRNA from independent pools of testis tissues isolated at equivalent stages of maturation as those used for sequencing. We performed qPCR to examine the expression of 12 genic-transcript-linked piRNAs (*piRNA 35*, *piRNA 275*, *piRNA 288*, *piRNA 622*, *piRNA 676*, *piRNA 697*, *piRNA 1258*, *piRNA 1452*, *piRNA 1524*, *piRNA 1551*, *piRNA 1588* and *piRNA 1948*) that were upregulated in the B groups (Fig. 4). As expected, all genic-transcript-linked piRNAs and their linked genes were highly expressed in the sexually mature stage, relative to the levels detected in the sexually immature group.

### The neighbor-joining tree analysis

**Figure 5:**
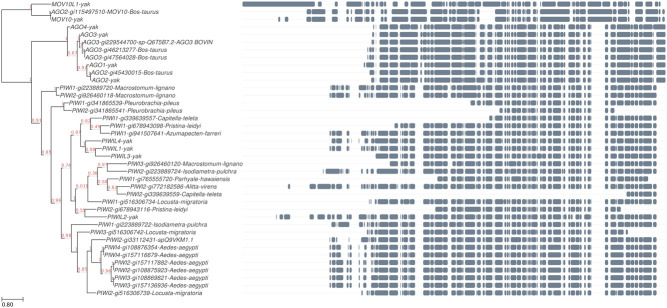
The neighbor-joining (NJ) tree of piRNA. NJ tree was built using the sequences of cattle, yak and other species and a bootstrap method. The value indicates the proportion of tree where a greater value indicates more reliable information with a maximum value of one, and a minimum value of zero. Bar = 80% nucleotide sequence divergence.

An individual phylogenetic tree was constructed based on partial piRNA sequences and concatenated by PIWI family sequences (Fig. 5). The resulting tree exhibited a typical branching pattern.

The data allowed identification of 39 *PIWI* genes, and the amino acid sequences of these genes belonging to different species were retrieved from the NCBI database. The amino acid sequences were used to construct the neighbor-joining (NJ) phylogenetic tree (Fig. 5). The sequence alignment revealed that these proteins exhibit high rates of variance in several amino acid positions. One possible cause of this variation is the occurrence of particular species.

### The expression levels of piRNA-related genes and proteins in testis tissues of yak

**Figure 6:**
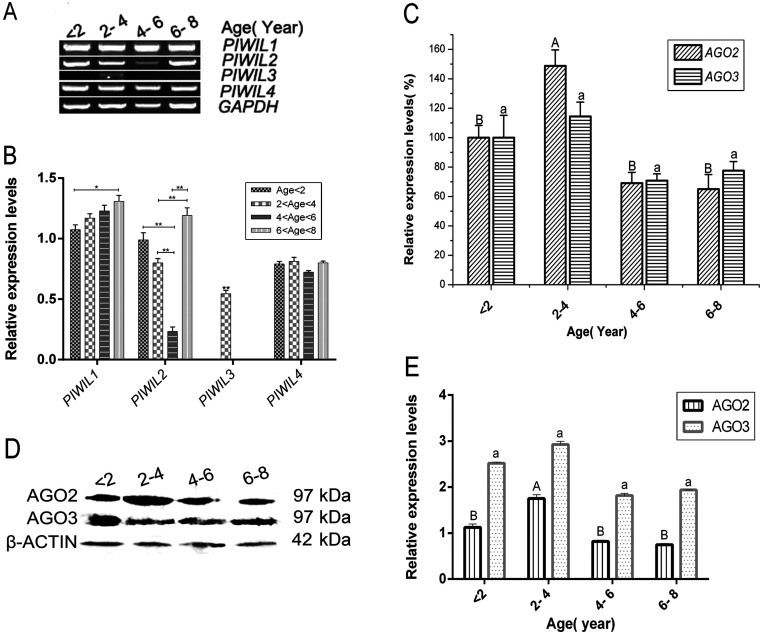
The expression of AGO2 and AGO3 from yak testis during postnatal development. (A) piRNAs expression profile determined by qRT-PCR for *Bos grunniens* testes samples from <2 year to 6–8 year Chinese Tibet plateau yak. (B) The *X*-axis represents genes and the *Y*-axis shows the relative expression levels of RNAs. Data shown are the mean ± s.e. of four groups. Statistical significance was determined by ANOVA followed by Tukey’s *post hoc* test, and different letters indicate the significant difference. *P ≤ 0.05, **P ≤ 0.01. (C) Data shown are the mean ± s.e. of four groups. Statistical significance was determined by ANOVA followed by Tukey’s *post hoc* test, different letters indicate significant difference (*P* < 0.05). (D) Western blot analysis of AGO2 protein and β-actin for samples from animals of different age. (E) The graph shows that the relative amount of AGO2 and AGO3. Data shown are the mean ± s.e. of four groups. Statistical significance was determined by ANOVA followed by Tukey’s *post hoc* test, and different letters indicate the significant difference (*P* < 0.05).

The sequence analysis indicated that the expression of the identified piRNAs and their linked genes was higher in germ cells than in other test samples (animal organ tissues), so we verified the expression of selected piRNA-linked genes, including *PIWI-like 1–4*. Testis piRNAs-like gene expression was compared among four different age stages using RT-PCR analyses and semi-quantitative evaluation (Fig. 6A). The expression of *PIWIL3* was significantly increased in sexually immature animals 2–4 years of age. In contrast, the expression of *PIWIL4* showed little variation among the different age groups. *PIWIL1* expression increased steadily with age. *PIWIL2* expression gradually decreased before age 6 years, but significantly increased in age 6–8 years after the animals reached sexual maturity (Fig. 6B).

In order to investigate the changes in the expression of the piRNAs in different development stages, *AGO2* and *AGO3* were analyzed by RT-qPCR. The analysis revealed that *AGO2* and *AGO3* mRNA levels were enhanced in testes from animals aged 2–4 years, compared to the levels detected in sexually mature animals. This effect was most obvious for *AGO2* expression (*P* < 0.05) (Fig. 6C). To examine if this increased mRNA expression resulted in higher protein expression of AGO2 and AGO3, we performed Western blot analysis on samples from the different age stages. As shown in Fig. 6D, AGO2 and AGO3 expression was significantly increased in animals aged 2–4 years in the sexual immature stage. In contrast, we detected no considerable elevation of expression levels for the animals in the 4–6 and 6–8 years age groups, compared to the sexually immature stages (Fig. 6E). The AGO3 protein level was slightly increased from age <2 to age 2–4 years.

### Immunohistochemical staining of AGO2 and AGO3 in yak testis

**Figure 7:**
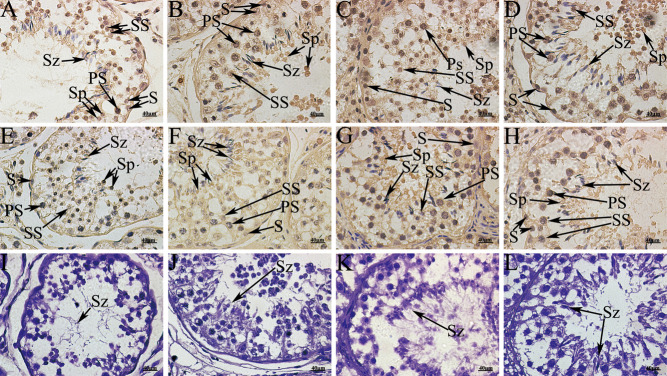
Immunohistochemistry with AGO2 and AGO3 using the immunoperoxidase method and hematoxylin counterstain from yak testis during postnatal development. (A, B, C and D) Expression of AGO2. (E, F, G and H) Expression of AGO3. (I, J, K and L) Negative control. (A, E and I) age <2 years; (B, F and J) 2 < age < 4 years; (C, G and K) 4 < age < 6 years; (D, H and L) 6 < age < 8 years. PS, primary spermatocytes; S, spermatogonia; Sp, spermatids; SS, secondary spermatocytes; Sz, spermatozoa.

To verify the presence of piRNA in spermatogenic cells, immunohistochemical analysis was performed (Fig. 7). Immunoreactivity of AGO2 and AGO3 was widely observed inside the seminiferous tubules, confirming the presence of these proteins in male germ cells.

The testis sample from animals aged <2 years showed that AGO2 was expressed at strong-to-moderate intensity in primary spermatocytes, and weakly in the spermatogonia and spermatids (Fig. 7A). AGO3 was expressed at weak-to-moderate intensity in secondary spermatocytes and was not detected in other cells (Fig. 7E). In the samples of animals aged 2–4 years, AGO2 was expressed with strong intensity in most primary and secondary spermatocytes (Fig. 7B). In the spermatids of these animals, AGO3 was expressed at various intensities and fractions, mostly in the nucleus, but also in the cytoplasm (Fig. 7F). The samples from animals older than 6 years showed wide expression levels of AGO2 in testicular germ cells (Fig. 7C and D) and strong expression of AGO3 in the spermatids, but weak-to-moderate expression in testicular germ cells (Fig. 7G and H). The immunohistochemical analyses indicated the presence of AGO2 and AGO3 in different cell types. Specifically, stronger immunoreactivity signal for AGO2 and AGO3 in the sperm tail region in spermatozoa and spermatids was observed.

The expression in the four age groups was quantitated as the average optical density value of AGO2 and AGO3 using ImagePro Plus6.0 Software. AGO2 was identified in all germ cells, as evidenced by positive cells. The signal for AGO2 in the 2- to 4-year-old group was significantly different from the other groups (*P* < 0.05) (Table 2). However, the AGO3 signal was not significantly different for the groups (*P* > 0.05) (Table 2).

**Table 2:** The optical density value of AGO2 and AGO3 in testis of yak.

**Age** (year)	**Integral optional density of AGO2**	**Integral optional density of AGO3**
<2	33,390.09 ± 7879.89^b^	25,767.83 ± 3270.81^a^
2–4	52,652.92 ± 4417.12^a^	34,358.10 ± 3839.82^a^
4–6	21,067.04 ± 2271.08^b^	23,382.87 ± 2465.34^a^
6–8	23,156.98 ± 1123.16^b^	30,016.12 ± 7224.55^a^

Significant difference between groups (*P* < 0.05). Data with a different letter (a, b) within the same line differ significantly at *P* < 0.05. Data with a same letter (a) within the same line differ significantly at *P* > 0.05.

## Discussion

PIWI proteins are specifically expressed in animal germ cells and are essential for germline development and gametogenesis. Recently, PIWI-interacting RNAs, a novel class of germ-cell-specific small non-coding RNAs, have been characterized as being associated with PIWI proteins (Rouget *et al.* 2010, Jiang *et al.* 2014, Roovers *et al.* 2015). This study represents one of the first characterizations of piRNA biology in the different stages of sexual maturity in yaks. The results presented here support potential roles for piRNAs in the classical control of transposon expression during spermatogenesis in the yak, as reported for other animals during gametogenesis (Rouget *et al.* 2010, Gou *et al.* 2014). The observed patterns of piRNA expression in yak testis is highly consistent with the result of recent studies (Liu *et al.* 2012, Ha *et al.* 2014) and supports the hypothesis that this regulatory pathway participates in transposon control in testis. Most importantly, we identified piRNAs in sexually immature yak testis that appear associated with target genes that are subsequently destined for reversal during sexual maturity.

In the present study, Illumina HiSeq technology was utilized to analyze the piRNA population produced in yak testis samples from four different age groups that included sexually immature and sexually mature animals. The read length distributions show clear peaks corresponding to miRNAs in the testes, and the testes show an additional prominent peak at the expected piRNA size of 28–32 nt (Brennecke *et al.* 2007, Russell *et al.* 2016). Interestingly, the peak of 24–27 nt present in the sexually immature testis reads may represent a population of shorter piRNAs.

The resulting reads of the appropriate size were aligned to the reference genome and cluster analysis was performed, revealing 331 clusters, a total length of 1,747,565 bp. From a step-by-step analysis of the clean reads, 6388 candidate piRNAs were isolated.

In addition, PIWI preferentially binds to piRNAs derived from the antisense strand of retrotransposon coding sequences that show a strong bias toward a uracil base (U) at the 5′ terminus (Ku & Lin 2014). This bias was present in all samples showing a Ping-Pong signature. Experiments investigating the source of the first U bias suggest that intrinsic properties of the PIWI protein partner are responsible for binding and stabilization of the first U containing piRNAs (Cora *et al.* 2014). The candidate piRNAs have a 75% U bias in the first base at the 5′ terminus, defining a strong Ping-Pong signature in them.

Many piRNA sequences are associated with transposable elements (TEs). Previous characterization showed that a main function of piRNA in the germ line is the silencing of TEs, and this role is highly conserved across animal species (Siomi *et al.* 2011*b*). We mapped piRNAs to known bovine and ancestral TEs and identified the various classes and their representation in each of our datasets. As expected, the most commonly targeted TE in our piRNA-rich datasets is the LINE element, LINE1. The abundance of piRNA with the potential to target LINE1 is likely due to the presence of 344,539 copies of LINE1, which comprises 11.78% of the yak genome. RTE repeats represent 9.24% coverage of the genome. Previous research has shown that piRNAs contribute to the activation of LINE and LTR retrotransposons in the male germ line, as well as the arrest of gametogenesis and complete sterility in males (Aravin *et al.* 2007, Carmell *et al.* 2007, Kuramochimiyagawa *et al.* 2008). However, LINE1 accounted for only 151 of our mapped piRNAs, less than LTR, which numbered 200. LINE1 was previously shown that the DNA methylation status of the regulatory regions in retrotransposons was regulated by PIWI family members during spermatogenesis. Taken together, these data suggest a proportional relationship between retrotransposon expression and potentially targeting piRNA. A more comprehensive characterization of TE and piRNA expression during gametogenesis will be crucial to understand more completely the effects on sperm development.

GO analyses of piRNA-generating genes showed enrichment in functions such as component organization or biogenesis and cellular, developmental, multicellular organismal, single-organism, biological adhesion, multi-organism, growth, reproductive and rhythmic processes.

To study the roles of protein–protein interactions in spermatogenesis, we selected 29 genes and found that they participated in a series of biological processes and signaling pathways, including the PI3K-Akt signaling pathway, focal adhesion, ECM–receptor interaction and processes related to Parkinson’s disease. PI3K-Akt is a serine/threonine protein kinase that functions as a critical regulator of cell survival and proliferation and plays important roles in the signaling pathways in response to growth factors and other extracellular stimuli to regulate several cellular functions (Song *et al.* 2005). The PPI analysis showed that UBB genes play important roles in the Parkinson’s disease pathway. However, this may be related to the time course of spermatogenesis. Sinnar and coworkers found that UBB could regulate fertility-related genes, which include a number of testis-specific or meiosis-specific genes (*Mtl5*, *Fabp9*, *Msh4*, *Spdya*, *Rec8* and *Zfp318*) (Sinnar *et al.* 2011). UBB-knockout mice have been found to have low-birth-weights and delayed development of spermatozoa (Sinnar *et al.* 2011). piRNAs are thought to be involved in gene silencing, specifically the silencing of transposons (2006). In mammals, it appears that the activity of piRNAs in transposon silencing is most important during the development of the embryo and spermatogenesis (Wang & Reinke 2008). Therefore, at different times, piRNAs may regulate the transposon of UBB and indirectly regulate the production of sperm.

To validate the piRNA expression pattern observed in the sequencing results, we adapted a Hairpin-it miRNA qPCR assay to detect piRNA from the sexually mature and immature groups. Based on the expression of 12 genic-transcript-linked piRNAs along with their linked genes, most of the piRNAs and piRNA-linked genes were more highly expressed in the sexually mature stage than in the sexually immature stage, suggesting that piRNAs are mainly expressed in spermatids. The observed expression pattern of the piRNAs was consistent with the detection of the largest number and the highest levels of piRNA in purified spermatocytes and round spermatids (Ro *et al.* 2007). piRNAs are thought to be required for germ cell development in vertebrate species (Morozova 2008). The majority of piRNA-linked genes may be functionally related to germ cells and testis in yaks and both piRNAs and piRNA-linked genes have shown major functions in the germ cells of vertebrate species (Thomson & Lin 2009). As shown in Fig. 4, we observed that PIWI family proteins show high evolutionary among with most vertebrate species. The chromosomal loci of the piRNA gene clusters are higher conserved in organisms such as mice, rats and humans. However, the conservation of piRNAs encoded sequence is non-conservation (Aravin *et al.* 2006*a*, Girard *et al.* 2006, Lau *et al.* 2006). Because of the RNA splicing activity of PIWI, AUB and AGO3 are different in various species with the evaluation, and which form complexes with piRNAs to carry out the function of inhibiting the regulation of transposons, transposons and retrotransposons and small-molecule RNA gene silencing. The expression of 12 genic-transcript-linked piRNAs and their linked genes was highest in the sexually mature group, possibly indicating that these piRNAs are produced from their linked genes to regulate various testis functions, but further investigation of the co-expression of piRNAs and their linked genes in testis is required to confirm this. piRNAs are defined by specific binding to the PIWI subfamily of AGO/PIWI family proteins (Lim *et al.* 2013). PIWI proteins are required for piRNA biogenesis and function and play a central role in germline development and gametogenesis (Thomson & Lin 2009). These functions may involve piRNAs and may function via RNA interference silencing complex (RISC)-mediated epigenetic and post-transcriptional regulation (Allshire 2002, Thomson & Lin 2009). We selected yak homologs of piRNA pathway genes, *PIWIL1–4*, *AGO2* and *AGO3*, for indirect functional validation of the putative piRNA-linked genes identified in the present study. We found that the *Piwil3 gene* exhibits expression in the 2- to 4-year stage. In addition, AGO2 and AGO3 are specifically expressed in the testis and are preferentially expressed at high levels in the testis of adult yaks during development. Levels of *PIWIL1* and *PIWIL2* were significantly increased in the testes of sexually mature males. These key genes control sexual maturation and are expressed earlier or at higher levels in yak development, which suggests an involvement of PIWI proteins in transcriptional gene silencing.

Immunohistochemical analyses of testicular samples from different age groups revealed that AGO2 and AGO3 bind to primitive germ cells, such as gonocytes and pre-spermatogonia in testis. Variable AGO2 and AGO3 affinity among germ cells and the loss of signal with age suggested that AGO2 bound strongly to spermatogonia, but only weakly to gonocytes during development. However, AGO3 bound to germ cells with moderate intensity during development, but was widely distributed in the testis.

The results indicate a potential role for the *PIWI* subfamily and piRNAs in translation control, which is consistent with the action of piRNAs as sequence-specific guides of PIWI proteins to regulate gene and transposon expression at the transcriptional and post-transcriptional levels (Yin & Lin 2007, Watanabe *et al.* 2011, Huang *et al.* 2013).

## Supplementary Material

Supporting Figure 1

Supporting Figure 2

Supporting Figure 3

Supporting Table 1

Supporting Table 2

Supporting Table 3

Supporting Table 4

## Declaration of interest

The authors declare that there is no conflict of interest that could be perceived as prejudicing the impartiality of the research reported.

## Funding

The research was supported by the grant from Chinese National 863 plan project (Project No. 2013AA102505-3).
