# Supplementary figures and images for: Identification and verification of potential piRNAs from domesticated yak testis
Source: Reproduction. 2017 Nov 3;155(2):117–27. doi: 10.1530/REP-17-0592 (PMC5763474; doi:10.1530/REP-17-0592)

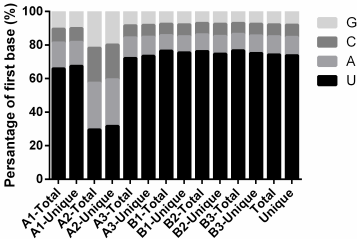

Supplement: Supporting Figure 1 [file rep-155-117-s001.pdf]

## The percentage of candidate piRNA first residue

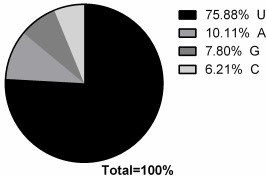

Supplement: Supporting Figure 2 [file rep-155-117-s002.pdf]

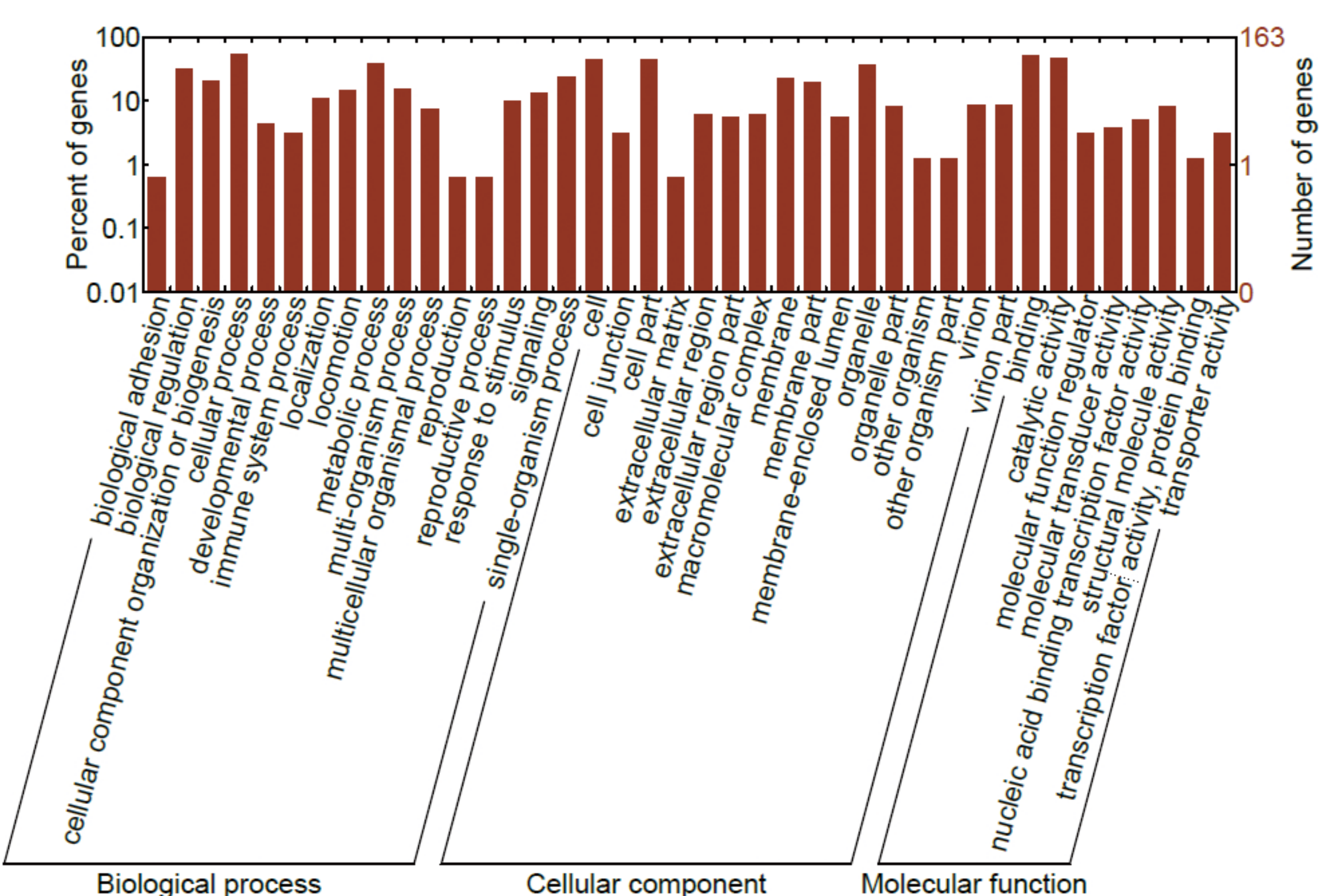

Supplement: Supporting Figure 3 [file rep-155-117-s003.pdf]
